# Predictors of return to work following motor vehicle related orthopaedic trauma

**DOI:** 10.1186/s12891-016-1019-6

**Published:** 2016-04-19

**Authors:** Darnel F. Murgatroyd, Ian A. Harris, Yvonne Tran, Ian D. Cameron, Darnel Murgatroyd

**Affiliations:** John Walsh Centre for Rehabilitation Research, The University of Sydney, Kolling Institute, Sydney, NSW Australia; South Western Sydney Clinical School, UNSW Australia, Liverpool, Sydney, Australia; Ingham Institute for Applied Medical Research, Sydney, Australia; South Western Sydney Local Health District, Liverpool, Sydney, Australia; John Walsh Centre for Rehabilitation Research, The University of Sydney, Kolling Institute, Royal North Shore Hospital, Pacific Hwy, St Leonards, NSW 2065 Australia

**Keywords:** Compensation and redress, Wounds and injury, Multiple trauma, Return to work

## Abstract

**Background:**

Work disability following motor vehicle related orthopaedic trauma is a significant contributor to the burden of injury and disease. Early identification of predictors for return to work (RTW) is essential for developing effective interventions to prevent work disability. The study aim was to determine the predictors (including compensation related factors) of time to RTW following motor vehicle related orthopaedic trauma.

**Methods:**

Admitted patients were recruited prospectively from two trauma hospitals with upper and/or lower extremity fractures following a motor vehicle crash. Baseline and follow up data were collected by written questionnaire. For baseline, this occurred in person within 2 weeks of injury. For follow up, this occurred by mail at six, 12 and 24 months. Additional demographic and injury-related information was retrieved from hospital databases. Analysis involved: descriptive statistics; logrank test to detect survival distributions of categorical variables; and Cox proportional hazards regression models for risks of time to RTW using baseline characteristic and compensation related variables (at 6 months).

**Results:**

Of 452 study participants 334 (74 %) were working pre-injury: results are based on this subset. Baseline characteristics were mean age 36 years (13.9 Standard Deviation [SD]), 80 % male; 72 % self-assessed very good-excellent pre-injury health, 83 % household income > AU$40,000 (Australian Dollar). Follow up data was available for 233 (70 %), 210 (63 %), and 182 (54 %) participants at six, 12 and 24 months respectively.

Significant risks of a longer time to RTW were greater injury severity, as measured by the New Injury Severity Score (NISS) (Hazards Rate Ratio [HRR] = 0.54, 95 % CI 0.35-0.82); and lower occupational skill levels (HRR = 0.53, 95 % CI 0.34-0.83). Significant risks of a shorter time to RTW were: recovery expectations for usual activities within 90 days (HRR = 2.10, 95 % CI 1.49-2.95); full-time pre-injury work hours (HRR = 1.99, 95 % CI 1.26-3.14); and very good self-assessed pre-injury health status (HRR = 1.41, 95 % CI 0.98-2.02). Legal representation (analysed at six months only) was not associated with time to RTW. At each time period, there were 146 (63 %), 149 (71 %), and 137 (76 %) working participants.

**Conclusions:**

A longer time to RTW was associated with greater injury severity and lower occupational skill levels; while a shorter time to RTW was associated with recovery expectations for usual activities within 90 days, full-time pre-injury work hours, and very good self-assessed pre-injury health status. Our findings reinforce existing research. There is an opportunity to trial interventions that address potentially modifiable factors. The issues surrounding legal representation are complex and require further research.

## Background

Work is pivotal to determining individual identity, social roles and status; it is also a key factor in physical and mental health; and it provides financial security and enables active participation in society [1]. Consequently, work disability following trauma intensifies the societal burden with increased disability, pain and health care utilisation rates [2–4]. In addition, motor vehicle related orthopaedic trauma is a significant contributor to the burden of injury and disease that commonly involves people of working age [5].

Early identification of predictors for return to work (RTW) after injury is essential as a prerequisite for developing effective interventions to prevent work disability and reduce the overall burden of injury [6]. However, there remains a lack of rigorous prospective studies following orthopaedic trauma that investigate these predictors. Results from a systematic review were inconclusive with limited predictors measured across studies, short follow up periods, and selective reporting of results [7]. Nonetheless, individual studies have shown that education, occupation, injury severity, self-efficacy and compensation related factors are predictive of work disability in this population [2–4, 8].

There are many predictors for RTW: individual worker factors such as job and injury characteristics; medical and vocational rehabilitation interventions; and organisational employer/insurer characteristics [6, 9]. Other factors include societal, legislative and macro-economic factors such as litigation, compensation scheme design, wage replacement benefits, and unemployment rates. Many of these are population specific [6].

In that context, there is evidence of an association between compensation related factors and poorer health outcomes, including RTW, following trauma [7, 10]. These associations have been found in workers compensation and traffic injury compensation systems across jurisdictions and injury types, despite the highly contextual socio-political environment in which compensation schemes operate. Similarly in qualitative research, adversarial claims processes, perceived illegitimacy of injury, and financial hardship have also impacted negatively on injury recovery and RTW [11–13].

Our study explored the association between individual worker and injury characteristics, compensation related factors, and time to RTW. Of particular interest were predictors that could be amenable to change [6, 7]. Thus, the aim was to determine the predictors (including compensation related factors) of time to RTW following motor vehicle related orthopaedic trauma.

## Methods

### Study design and setting

The inception cohort study recruited patients from two trauma hospitals in Sydney, New South Wales (NSW), Australia between November 2007 and February 2011, to provide a representative sample of motor vehicle related orthopaedic trauma requiring inpatient hospitalisation. Eligible patients identified via a hospital trauma database were invited to participate. Informed consent was obtained. Patients from Culturally and Linguistically Diverse (CALD) backgrounds were interviewed with an English speaking family member.

Inclusion criteria were:

- admission to hospital within 2 weeks of injury;
- involvement in a motor vehicle crash;
- age 18 years or over; and
- an upper or lower extremity fracture (humerus, radius, ulna, pelvis, acetabulum, femur, patella, tibia, fibula, talus, calcaneus).

All extremity and pelvic fractures that required admission to hospital were included. These fractures were selected because treatment usually involves hospital admission and surgical intervention, but surgery alone was not an inclusion criterion. There were no restrictions, therefore, intra-articular and/or extra-articular, open and/or closed, and simple and/or complex fractures were included. Spinal trauma was excluded because these injuries were not usually treated at the participating centres.

Exclusion criteria were:

- dementia or a significant pre-existing cognitive impairment preventing the ability to consent;
- spinal cord injury;
- Glasgow Coma Score <12 on admission;
- amputation of a limb; or
- isolated clavicle, scapula, phalangeal, carpal, metacarpal, tarsal or metatarsal fractures not requiring admission to hospital.

There were 32 variables: allowing for 10 participants per variable, a sample size of 450 was calculated [14]. This was considered sufficient to accommodate a 25 % loss to follow up, based on similar research [15].

Follow up questionnaires were posted at six, 12 and 24 months post injury. If no response was received by 3 weeks, up to six attempts were made to contact participants by telephone and/or by mailing additional questionnaires.

Baseline data were collected in hospital within 2 weeks of injury using a written questionnaire. Demographic and injury related information was retrieved from the hospital trauma database and records. The study factors were chosen to reflect the study aims with reference to relevant research [16–18]. The study was approved by the governing human research ethics committees (South Western Sydney Local Health District, South Eastern Sydney Local Health District, and The University of Sydney).

### Injury related factors

Injuries were coded using the Abbreviated Injury Scale (AIS) (1990 Revision, Update 98) [19]. The AIS ranks injuries from one to six (six is not survivable). The Injury Severity Score (ISS) and New Injury Severity Score (NISS) were calculated by summing the squares of the three highest AIS scores from different body regions (ISS), and regardless of body region (NISS). They are indicators of potential mortality [20]. Injuries were classified as minor – moderate [1–8], serious [9–15] or severe – critical (16–75) [21].

### Socio-demographic factors

Socio-demographic factors included age, gender, marital status, occupation, and education. Income was measured exclusive and inclusive of household structure to allow for potential differences in income distribution. An adjusted income (inclusive of household structure) was calculated by dividing the income by the sum of points: 1 for the first person aged ≥15 years; 0.5 for each additional person aged ≥15 years; and 0.3 for each person aged <15 years [22].

### Health related factors

Self-reported chronic illnesses were measured as an indicator of baseline health status, they were asthma, cancer, heart and circulatory conditions, diabetes, arthritis, osteoporosis, mental and behavioural problems, and neck/back disorders. These self-reported illnesses were compatible with the National Health Priority Areas initiative (conditions that imposed high social and financial costs on Australian society) [23]. A chronic condition was defined as one which the patient currently has, and which has lasted or is expected to last for six months or more, from the Australian Bureau of Statistics (ABS) Health Survey [22, 23]. Other factors included: recent injuries (other than the motor vehicle crash) in the last 4 weeks requiring medical intervention or a decrease in usual activities; medication use in the last 2 weeks for a chronic illness; and smoker status [22].

Previous research has found an association between poor expectations for recovery and poor RTW and/or health outcomes, but there was an absence of validated measures [9, 18, 24, 25]. Therefore, we used two applicable measures from a large Canadian study of soft tissue injuries [24]. The questions asked were: If you were working before the motor vehicle accident, do you think you will recover enough to return to your usual job (Y/N); and How long do you think it will take for you to return to your usual activities (number of days).

Alcohol consumption was measured using the first three questions of the Alcohol Use Disorders Identification Test: Self-Report Version (AUDIT-C) [26]. The word ‘standard’ and ‘in the past year’ were added. Risk of long/short term harm due to alcohol consumption was assessed with the National Health and Medical Research Council (NHMRC) levels [27]. Because these levels were mismatched with the AUDIT-C categories, an algorithm was used based on the Bettering the Evaluation of Care and Health (BEACH) Survey, (Associate Professor K Conigrave, personal communication March 19, 2007). Categories for other study factors are explained in the Tables.

### Compensation related measures

The majority of compensation related factors were recorded at six months because most questions would have been unanswerable at baseline. The following questions were asked: claim made (Y/N); claim type (Compulsory Third Party [CTP]/Workers Compensation [WC]/other); claim accepted (Y/N/don’t know); and legal representation obtained (Y/N). Claim made ‘Yes’ was defined as making a personal injury claim of any type; which included a CTP Accident Notification Form (ANF) for expenses less than AU$5,000 (Australian Dollar) within 28 days of injury. At baseline self-reported fault of the driver was measured (i.e. whether the driver considered that they caused the crash). Passengers and pedestrians were considered not at fault.

In NSW, CTP personal injury insurance is a privately underwritten, statutory, modified common law scheme. All motor vehicles travelling on public roads must be registered and insured for CTP. A CTP claim is made against the owner or driver of the vehicle at fault. Since April 2010, regardless of who was at fault, anyone injured in a motor vehicle crash can access limited entitlements (medical expenses and lost wages up to AU$5,000). The WC scheme is publically underwritten with statutory benefits and administered by private insurers. To make a claim for injury the motor vehicle crash must have occurred during travel between place of employment, home and/or any work-related place and a person injured (regardless of fault). Further, the insurer must be notified of an injury within 48 hours and there is a legal obligation under the NSW WC legislation for employers to accommodate RTW of an injured employee, although there is no obligation under the NSW CTP legislation [28, 29]. In 2015, the government regulators of these schemes merged to form the State Insurance Regulatory Authority (SIRA).

For both schemes, a claim must be lodged within six months of injury and the insurer has three months to determine final liability (accept or deny the claim). Provisional acceptance of liability enables earlier payment for medical expenses, and for WC weekly wage benefits based on work capacity and weeks since injury [29]. In CTP, lump sum payments are available on a case-by-case basis for financial hardship. Entitlements include past and future losses across each scheme (e.g. medical expenses, loss of income, and pain and suffering/impairment) [28, 29]. Legal representation can also be obtained at any time for either scheme.

### Outcome measure - return to work

There are no standardised measures for RTW. Those used in this study encapsulated self-reported duration and level of work [6, 7]. The primary measure was time (days) to return to work (i.e. from date of injury to date of RTW). At each time period work status (Y/N) was measured. Working participants were then asked the date of RTW, if they were working full/modified duties (e.g. lifting restrictions), and full-time (usually working at least 35 hours per week) or part-time (usually working 1–35 hours per week) [30]. These questions were asked pre-injury (baseline) and post-injury (six, 12 and 24 months). Participants were also asked if their inability to RTW was crash-related, and if they had changed their occupation following injury.

### Data analysis

RTW baseline characteristics, including full/modified duties and full/part-time, were summarised using descriptive statistics. Outcomes were assessed using survival analysis with Cox proportional hazards regression models employed to determine the multivariate predictors of time to RTW. The Cox model is considered an appropriate approach to accounting for time to an event [31]. The variables selected for the model have been shown to be independent predictors of RTW and/or potential confounders of poorer outcomes in other research [7, 16–18]. Similarly, compensation-related factors were selected for the same reasons [2, 4, 7, 8].

Selection of variables for the Cox model was based on associations between baseline characteristics, including compensation related factors and time to RTW. These were assessed using the logrank test to detect differences in the survival distributions across categorical variables. All variables with p-value ≤ 0.20 were entered into the Cox regression model using a backward elimination process with an entry p-value < 0.05 and an exit p-value < 0.10. Variable selection was confirmed through explained variation and predictive accuracy using R-squared values calculated with the Cox and Snell R-squared approach and a concordance index [32]. The concordance index is a widely applicable measure with progressive addition of factors that improve discrimination of the model. When a variable is added and the c-index plateaus or decreases, that variable and additional variables can be regarded as noise and excluded to avoid over fitting in the model [31]. Furthermore, explained variance using R-squared describes the relative importance of adding each variable into the Cox regression model.

Data from participants where the endpoint (RTW) had not occurred or was unknown at 24 months were considered censured. In studies of survival, in which the outcome is death, Hazard Rate Ratios (HRR) greater than 1 indicates risk. However, in this study, the fewer cumulative days of time taken to RTW, the more positive the outcome, in terms of injury recovery/RTW, and the higher the HRR. Therefore, a HRR less than 1 indicates higher risk and a longer time taken to RTW. A test of proportionality was performed on all predictors, and claim made and legal representation. The assumption of proportionality was not violated (*p* > 0.05) [33].

A separate Cox proportional hazards regression analysis was done for compensation related variables (claim made and legal representation), as these variables were measured at the six month time point and only a portion of participants made a claim and sought legal representation. In this Cox regression, claim made and legal representation were added to the final variables in the baseline RTW model. All data analysis was performed using SPSS statistical software version 22 (SPSS Inc, USA).

## Results

**Fig. 1:**
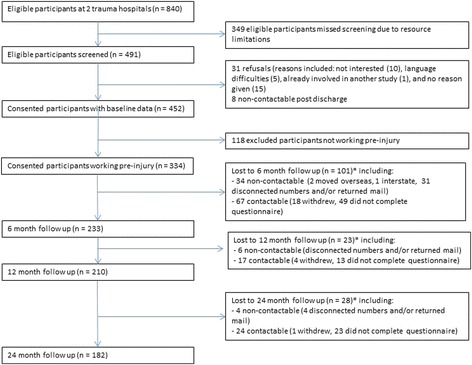
Flow chart of study participants

From November 2007 to February 2011, 840 eligible participants were admitted to hospital across both sites, 491 were screened (349 eligible participants missed being screened due to resource limitations), and 452 (92 %) consented to participate. There were 31 refusals and eight who were discharged and unable to be contacted. Additional information about recruitment and follow up for study participants is shown in Fig. 1. There were significant differences (*p* < 0.05) in baseline characteristics, namely socio-demographic and socio-economic factors, between those working and not working pre-injury. These differences were expected and people not working were not included in the analyses (data not shown). Of the 452 participants, our subsequent results are based on the subset of 334 (74 %) participants who worked up to the time of injury.

### Baseline characteristics

Baseline characteristics were: mean age 36 years (13.9 Standard Deviation [SD]); 80 % male; 72 % self-assessed very good-excellent pre-injury health; 83 % annual household income > AU$40,000. Follow up data was available for 233 (70 %), 210 (63 %), and 182 (54 %) participants at six, 12 and 24 months respectively. There were significant differences between responders and non-responders at six, 12 months and 24 months; this is explained in Table 1. For all other variables there was no significant difference (p > 0.05) (data not shown). In addition, there were significant differences between those participants that made a claim at six months (*n* = 140) and those that did not (*n* = 91). This reflected eligibility to claim under the NSW legislation: those participants more likely to make a claim were not at fault (78 %), had crashed on a public road (94 %), and worked pre-injury (77 %). For all other variables there was no significant difference (p > 0.05) (data not shown).

**Table 1:** Baseline characteristics and health status of participants in the study compared to non-participants at six, 12 and 24 month follow up

	Participation at six months			Participation at 12 months			Participation at 24 months		
Variable	No (*n* = 101)	Yes^a^ (*n* = 233)	P	No (*n* = 124)	Yes^a^ (*n* = 210)	P	No (*n* = 151)	Yes^a^ (*n* = 182)	p
Age (years), Mean (SD)	31.9 (12.0)	38.2 (14.2)	**	31.7 (11.5)	39.0 (14.4)	**	31.5 (11.7)	40.3 (14.3)	**
New Injury Severity Score, No. (%)			NS			*			NS
Minor - moderate 1-8	22 (21.8)	41 (17.6)		27 (21.8)	36 (17.1)		33 (21.9)	30 (16.5)	
Serious 9-15	50 (49.5)	94 (40.3)		61 (49.2)	83 (39.5)		71 (47.0)	73 (40.1)	
Severe - critical 16-75	29 (28.7)	98 (42.1)		36 (29.0)	91 (43.3)		47 (31.1)	79 (43.4)	
Marital status, No. (%)			**			**			**
Single	60 (60.0)	87 (37.5)		68 (54.8)	79 (38.0)		86 (57.0)	61 (33.9)	
Married/defacto	36 (36.0)	131 (56.5)		53 (42.7)	114 (54.8)		61 (40.4)	105 (58.3)	
Divorced/widowed	4 (4.0)	14 (6.0)		3 (2.4)	15 (7.2)		4 (2.6)	14 (7.8)	
Occupation skill level^b^, No. (%)			*			NS			*
Managers/professionals	20 (19.8)	58 (25.0)		23 (18.5)	55 (26.3)		60 (26.1)	38 (17.1)	
Tradespersons	26 (25.7)	85 (36.6)		36 (29.0)	75 (35.9)		71 (30.9)	55 (24.8)	
Intermediate clerical	17 (16.8)	35 (15.1)		21 (16.9)	31 (14.8)		27 (11.7)	37 (16.7)	
Elementary related	38 (37.6)	54 (23.3)		44 (35.5)	48 (23.0)		53 (23.0)	72 (32.4)	
Body Mass Index (BMI)^c^ (kg/m^2^)			*			NS			NS
<18.50 (underweight)	4 (4.0)	2 (0.9)		4 (3.2)	2 (1.0)		4 (2.6)	2 (1.1)	
18.50-24.99 (normal)	45 (44.6)	78 (33.8)		50 (40.3)	73 (35.1)		64 (42.4)	58 (32.2)	
≥25.00 (overweight)	35 (34.7)	89 (38.5)		47 (37.9)	77 (37.0)		53 (35.1)	71 (39.4)	
≥30.00 (obese)	17 (16.8)	62 (26.8)		23 (18.5)	56 (26.9)		30 (19.9)	49 (27.2)	
Smoking history, No. (%)			*			NS			NS
Current smoker	38 (38.0)	51 (22.0)		40 (32.5)	49 (23.4)		48 (32.0)	40 (22.1)	
Ex-smoker	23 (23.0)	67 (28.9)		32 (26.0)	58 (27.8)		36 (24.0)	54 (29.8)	
Never smoked	39 (39.0)	114 (49.1)		51 (41.5)	102 (48.8)		66 (44.0)	87 (48.1)	
Self-reported chronic illnesses (yes) No. (%)	17 (16.8)	76 (32.6)	**	26 (21.0)	67 (31.9)	*	35 (23.2)	58 (31.9)	NS
Medication use (current), No. (%)	11 (10.9)	56 (24.1)	**	13 (10.5)	54 (25.8)	**	19 (12.6)	48 (26.5)	**
Vehicle type, No. (%)			*			*			*
Motor vehicle	61 (60.4)	114 (48.9)		71 (57.3)	104 (49.5)		87 (57.6)	88 (48.4)	
Motorcycle	31 (30.7)	109 (46.8)		42 (33.9)	98 (46.7)		53 (35.1)	87 (47.8)	
Bicycle	9 (8.9)	10 (4.3)		11 (8.9)	8 (3.8)		11 (7.3)	7 (3.8)	

^a^Participation status ‘yes’ was measured using the information recorded in variables - work status at six, 12 and 24 months, and the Short Form-36 Version 2.0 (SF36v2), Physical Component Score (PCS) at six, 12 and 24 months respectively

** *P* < 0.01, *P < 0.05, *NS* not significant

^b^The measure for occupation is from the Australian Standard Classification of Occupations (ASCO), Cat. No. 1220.0, Australian Bureau of Statistics 1997. See Table 2, Occupational skill level for all categories

^c^BMI classification is from the Global Database on Body Mass Index, World Health Organisation

### Characteristics of return to work

At baseline, of the 334 who worked, 83 % were full-time and 96 % performed full duties. At six months, of the 146 (63 % of responders) who worked, 65 % were full-time and 64 % performed full duties. At 12 months, of the 149 (71 % of responders) who worked, 73 % were full-time and 69 % performed full duties. At 24 months, of the 137 (75 % of responders) who worked, 81 % were full-time and 79 % performed full duties. In addition, at six months, failure to RTW was related to the crash for 84 %, and 10 % had changed occupation. At 12 months, failure to RTW was related to the crash for 80 %, and 16 % had changed occupation. At 24 months, failure to RTW was related to the crash for 81 %, and 22 % had changed occupation.

Overall, there were nine participants who initially returned to work at either six or 12 months but did not remain at work during the subsequent follow up period(s). Of these, five participants worked at six and 12 months but not at 24 months. Two participants returned to work at 12 months but no longer worked at 24 months, and two participants who had returned to work at six months no longer worked at 24 months.

### Predictors of time to return to work

**Fig. 2:**
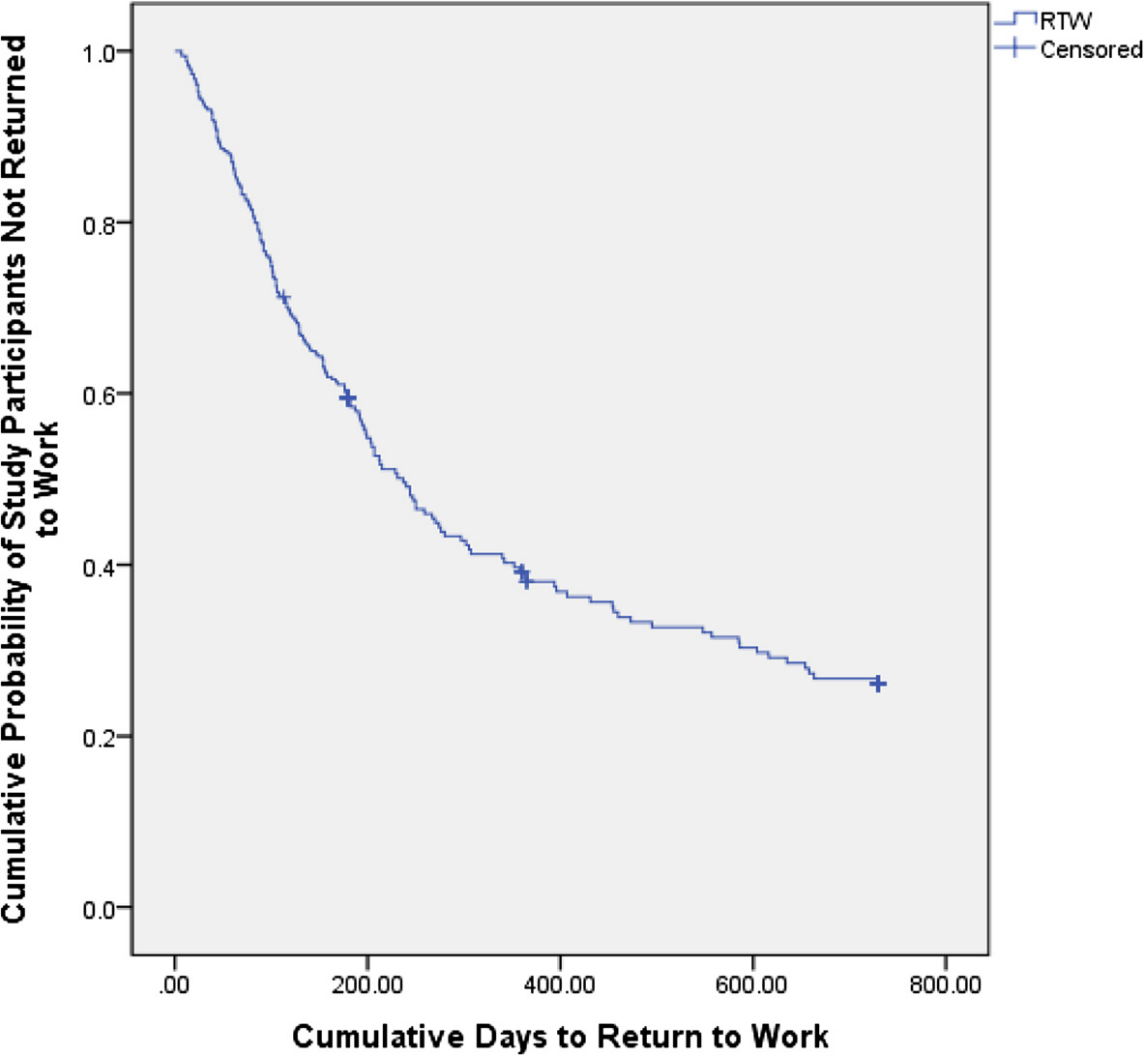
Kaplan-Meier estimate of the cumulative time (days) to return to work for study participants (*n* = 334)

For all 334 study participants, the median time to RTW was 231 days (95 % CI 190.05-271.95). For RTW, the probability of participants working at six months was 40.6 %, at 12 months was 62.2 %, and at 24 months was 74.2 %. This is based on the Kaplan-Meier estimates of the survival curve as shown in Fig. 2. Associations between baseline characteristics and time to RTW are shown in Table 2. The significant variables identified in the logrank test, including age and sex, were entered into the Cox proportional hazards regression model. Based on the variables identified from the backwards elimination process, Table 3 shows the concordance (c-index) and R-squared of each of the variables as they were added to the Cox model. The c-index plateaued at the variable of smoking history; the remaining variables were not included in the model. Of the variables that were not significant only age and sex were deemed necessary to be included in the Cox model.

**Table 2:** Baseline characteristics and time to RTW of study participants (*n* = 334)

Variable	No. (%)	Median time to RTW	Logrank Test *p* value
Age	334		
Injury Severity Score			0.09
Minor - moderate 1-8	84	204	
Serious 9-15	198	240	
Severe - critical 16-75	52	259	
New Injury Severity Score			0.002
Minor - moderate 1-8	63	204	
Serious 9-15	144	203	
Severe - critical 16-75	127	305	
Index of Relative Socioeconomic Disadvantage^a^			0.57
Most disadvantaged	89	215	
Disadvantaged	34	365	
Average	64	270	
Advantaged	78	207	
Most advantaged	69	198	
Sex			0.69
Female	67	199	
Male	267	244	
Marital status			0.47
Single	147	244	
Married/de facto	167	212	
Divorced/widowed/separated	18	270	
Education skill level^b^			0.13
Bachelor degree and above	60	138	
Certificate and advanced diploma	143	213	
Secondary education	118	305	
Pre-primary and primary education	10	229	
Occupation skill level^b^			0.01
Managers/administrators/professionals/associate professionals	78	125	
Tradespersons/advanced clerical and service workers	111	229	
Intermediate clerical/sale/service production/transport workers	52	396	
Elementary clerical/sales/service/labourers/related workers	92	340	
Work level before injury			0.87
Full Duties	321	231	
Part Duties	13	213	
Work hours before injury^c^			0.14
Full-time	273	215	
Part-time	57	302	
Pre-injury job satisfaction^d^			0.28
Satisfied	320	231	
Not Satisfied	14	342	
Recovery expectations for work			0.08
Yes	298	212	
No	32	280	
Recovery expectations for usual activities			<0.001
≤90	202	177	
>90	111	455	
Language other than English			0.08
Yes	108	250	
No	226	231	
Total yearly household income^e^ (before tax, AU) excluding number of people in household			0.17
≤$39,999	53	302	
$40,000-$79,999	110	250	
≥$80,000	148	194	
Total adjusted yearly household income^e^ (before tax, AU) including number of people in household			0.47
≤$39,999	153	240	
$40,000-$79,999	121	250	
≥$80,000	37	154	
Body Mass Index (BMI)^f^ (kg/m^2^)			0.54
<18.50 (underweight)	6	407	
18.50-24.99 (normal)	123	213	
≥25.00 (overweight)	124	203	
≥30.00 (obese)	79	302	
Smoking history			0.02
Current smoker	89	394	
Ex-smoker	90	207	
Never smoked	153	199	
Self-reported chronic illnesses			0.88
Yes	93	215	
No	241	240	
Medication use			0.93
Yes	67	229	
No	266	237	
Recent injury other than crash			0.40
Yes	16	365	
No	316	231	
Risk of long term harm due to alcohol consumption^g^ (standard drinks^h^/week)			0.92
Low risk - ≤28 male or ≤14 female	311	231	
Risky - 29–42 male or 15–28 female	13	358	
High risk - ≥43 male or ≥29 female	9	244	
Risk of short term harm due to alcohol consumption^g^ (yes)			0.14
Yes	116	297	
No	218	215	
Self-reported at-fault			0.04
Yes	125	203	
No	208	250	
Vehicle type			0.02
Motor vehicle	175	276	
Motorcycle	140	199	
Bicycle	19	182	
Pre-morbid neck pain in last 6 months			0.70
Yes	15	203	
No	319	237	
Post-morbid neck pain			0.74
Yes	59	215	
No	275	231	
Crash on a public road			0.06
Yes	297	240	
No	37	156	
Self-assessed pre-injury health status^ij^,			0.03
Excellent	103	240	
Very good	137	199	
Good	78	250	
Fair-Poor	16	-	
Claim made by 6 months			0.08
Yes	140	178	
No	91	120	
Legal representation at 6 months			0.007
Yes	95	199	
No	136	122	

^a^The Index of Relative Socioeconomic Disadvantage (IRSD) is a summary measure of economic and social conditions within a particular area/postcode (e.g. employment, fluency in English and household size). It is taken from the Census of Population and Housing: Socio-Economic Indexes for Areas (SEIFA), Cat no. 2039.0.55.001: Australian Bureau of Statistics; 2001. A low score is indicative of greater socioeconomic disadvantage

^b^Measures for occupation and education are from the Australian Standard Classification of Occupations (ASCO), Cat. No. 1220.0, Australian Bureau of Statistics 1997 and the Australian Standard Classification of Education (ASCED), Cat. No. 1272.0, Australian Bureau of Statistics 2001

^c^Measures for full-time (usually working at least 35 hours per week) and part-time (usually working 1–35 hours per week) are from the Australian Health Survey: Users' Guide, 2011–13, Cat. No. 4363.0.55.001, Australian Bureau of Statistics

^d^Pre-injury job satisfaction is based on the stem question from the Measure of Job Satisfaction questionnaire by Traynor, M. and Wade, B. 1993

^e^Categories of income are from the Household, Income and Labour Dynamics in Australia (HILDA) Survey Wave 6 Household Questionnaire

^f^BMI classification is from the Global Database on Body Mass Index, World Health Organisation

^g^Questions to determine risk of harm were from the Alcohol Use Disorders Identification Test: Self-Report Version (AUDIT-C) were resourced from the Drink-less program, The University of Sydney. http://sydney.edu.au/medicine/addiction/drinkless/resources.php

^h^1 standard drink contains 12.5 millilitres or 10 grams of alcohol according to the National Health and Medical Research Council (NHMRC), Australian Alcohol Guidelines Health Risks and Benefits, October 2001

^i^Self-assessed health status is based on Question 1 from the Short Form-36, Version 2.0, (SF36v2)

^j^No median score for fair-poor self-assessed pre-injury health status, the median indicates that more than half did not return to work (mean = 529 days)

**Table 3:** Concordance (c-index), R squared as each variable is added into the model

Factor	R-squared	C-index
Recovery expectations for usual activities	0.087	0.605
Occupation skill level^a^	0.104	0.643
New injury severity score	0.121	0.656
Self-assessed pre-injury health status^b^	0.154	0.662
Work hours before injury^c^	0.177	0.671
Smoking history	0.194	0.678
Education skill level^a^	0.197	0.679
Recovery expectations for work	0.206	0.687
Injury severity score	0.212	0.687
Total yearly household income^d^	0.225	0.695
Self-reported at fault	0.227	0.695
Language other than English	0.240	0.699
Crash on public road	0.241	0.700
Risk of short term harm due to alcohol consumption^e^	0.244	0.700
Age	0.251	0.704
Sex	0.252	0.707

The blank row indicates the point where the concordance index plateaus Factors above this were maintained while factors below this were dropped

^a^Measures for occupation and education are from the Australian Standard Classification of Occupations (ASCO), Cat. No. 1220.0, Australian Bureau of Statistics 1997 and the Australian Standard Classification of Education (ASCED), Cat. No. 1272.0, Australian Bureau of Statistics 2001

^b^Self-assessed health status is based on Question 1 from the Short Form 36, version 2, (SF36v2)

^c^Measures for full-time (usually working at least 35 hours per week) and part-time (usually working 1–35 hours per week) are from the Australian Health Survey: Users' Guide, 2011–13, Cat. No. 4363.0.55.001, Australian Bureau of Statistics

^d^Categories of income are from the Household, Income and Labour Dynamics in Australia (HILDA) Survey Wave 6 Household Questionnaire.

^e^Questions to determine risk of harm were from the Alcohol Use Disorders Identification Test: Self-Report Version (AUDIT-C) were resourced from the Drink-less program, The University of Sydney. http://sydney.edu.au/medicine/addiction/drinkless/resources.php

The Cox proportional hazards regression model for risks of time to RTW is presented in Table 4. The significant risks of taking a longer time to RTW were: greater injury severity (NISS), namely those with severe-critical injuries as compared to those with minor-moderate and serious injuries, and lower occupational skill levels as compared to managerial or professional skill levels. In the same model, the significant risks of taking a shorter time to RTW were: full-time pre-injury work hours compared to part-time pre-injury work hours; recovery expectations for usual activities of ≤90 days compared to recovery expectations for usual activities of ≥90 days, and having very good self-assessed pre-injury health status as compared to having excellent self-assessed health status.

**Table 4:** Cox proportional hazards regression model for predictors of time (days) to RTW

Factor	B	SE	P value	HRR	95 % CI
Age	0.009	0.006	0.10	1.010	0.998–1.021
Sex (Male)	−0.038	0.202	0.851	0.963	0.648–1.430
New Injury Severity Score			0.007		
Minor - moderate 1-8	-	-	-	-	-
Serious 9-15	−0.170	0.202	0.401	0.844	0.568–1.254
Severe - critical 16-75	−0.619	0.216	0.004	0.539	0.353–0.822
Occupation skill level^a^			0.05		
Managers/administrators/professionals/associate professionals	-	-	-	-	-
Tradespersons/advanced clerical and service workers	−0.355	0.204	0.081	0.701	0.470–1.045
Intermediate clerical/sale/service production/transport workers	−0.311	0.249	0.211	0.733	0.450–1.193
Elementary clerical/sales/service/labourers/related workers	−0.632	0.227	0.003	0.532	0.341–0.829
Work hours before injury (Full-time)^b^	0.688	0.232	0.003	1.989	1.261–3.136
Recovery expectations for usual activities (≤90 days)	0.741	0.174	<0.001	2.099	1.494–2.949
Self-assessed pre-injury health status^c^			0.005		
Excellent	-	-	-	-	-
Very good	0.343	0.184	0.062	1.409	0.983–2.019
Good	−0.111	0.219	0.612	0.895	0.582–1.376
Fair-Poor	−1.029	0.476	0.031	0.357	0.141–0.908
			0.090		
Current smoker	-	-	-	-	-
Ex-smoker	0.368	0.222	0.097	1.445	0.936–2.232
Never smoked	0.434	0.201	0.031	1.543	1.041–2.288

^a^Measures for occupation and education are from the Australian Standard Classification of Occupations (ASCO), Cat. No. 1220.0, Australian Bureau of Statistics 1997

^b^Measures for full-time (usually working at least 35 hours per week) and part-time (usually working 1–35 hours per week) are from the Australian Health Survey: Users' Guide, 2011–13, Cat. No. 4363.0.55.001, Australian Bureau of Statistics

^c^Self-assessed health status is based on Question 1 from the Short Form-36, Version 2.0, (SF36v2)

In terms of compensation related factors, overall 140/231 (60 %) made a claim at six months (there was missing data for 2/233 responders for the compensation related questions at six months). Of those who made a claim, 95/140 (68 %) sought legal representation at six months. Making a claim at six months was not associated with time to RTW, the HRR was 0.89 (95 % CI 0.60-1.33). The logrank analysis of seeking legal representation at six months was associated with a longer time to RTW (see Table 2). However, in the Cox regression with baseline variables included, legal representation was not associated with time to RTW; the HRR was 0.81 (95 % CI 0.54-1.21).

## Discussion

Time to RTW was associated with both injury and non-injury related factors in a cohort with motor vehicle related moderate-severe orthopaedic injuries. The main findings were that greater injury severity and lower occupational skill levels were significant risks of a longer time to RTW. Whereas, recovery expectations for usual activities of ≤90 days, full-time pre-injury work hours, and very good self-assessed pre-injury health status were significant risks of a shorter time to RTW. Legal representation at six months was not associated with time to RTW.

### Predictors of time to return to work

In our study, the significance of injury severity as a risk of time to RTW was driven by those with severe-critical injuries (ISS 16–75). Similarly, the significance of occupation was driven by those with lower occupational skill levels such as elementary workers and tradespersons. Existing research confers that injury severity and occupational skill level are predictors of RTW, particularly for lower limb injuries [3, 4, 8, 34, 35]. This result is not unforeseen given the socio-demographic profile of the cohort – mean age 36 years, 80 % male, and 33 % tradespersons, advanced clerical or service workers. Adequate physical function is likely to be an important component of work. In other research, these factors have been independent predictors of RTW, although the level of evidence is variable [7]. Likewise, the significance of full-time pre-injury work hours and very good pre-injury health status is likely to be dependent on the study population. These factors have been reported as predictors of a shorter time to RTW and recovery [4, 7, 16, 36]. Again, results are inconsistent and measures vary. For example, higher baseline income or job involvement is measured instead of pre-injury work hours [4, 7].

Recovery expectations and illness perception, and low self-efficacy, are significant predictors of RTW rates and/or recovery across a range of injuries and illnesses [7, 24, 25, 37, 38]. This shows they are robust predictors of RTW that relate to the individual rather than a specific diagnosis. These predictors are complex and multidimensional [24, 37, 39]. Theoretically, self-efficacy (i.e. person’s belief in their own competence) materialises during childhood and evolves throughout life. Those with strong self-efficacy master problems, recovering expeditiously; those with weak self-efficacy avoid challenges, focusing on negative outcomes [40]. Similarly, illness perception is based on a self-regulatory model that appraises a person’s response to their illness event [41]. In other words, how well you think you will recover can influence how well you actually recover.

The association between making a claim and legal representation, and poor RTW rates or recovery is well documented [7, 10]. As before, results vary according to the study population, outcome measures, and possibly the compensation scheme. In this study, the logrank test between legal representation and time to RTW was significant. However, baseline variables in the Cox regression could be a common cause of legal representation and time to RTW. In addition, it may be that people seek legal representation because they haven’t returned to work and/or because of other intervening factors, or those who seek legal representation are more likely to take longer to RTW. It is not possible to address these issues in this study. Regardless, there was no association once baseline variables were taken into account.

More recently, within a compensable setting, legal representation has been linked to socio-economic and psychosocial factors such as: stressfulness of making a claim; poorer baseline mental health; higher disability; socio-economic disadvantage; and financial entitlements [42–44]. This suggests that people seeking legal representation have different characteristics compared to other compensable and non-compensable participants; which could be partly or wholly responsible for prolonging their RTW.

Other research shows that people seek legal advice to help with the adversarial claims processes, communication and administrative deficits with insurers, perceived illegitimacy of their injury, and accessing reasonable entitlements [11–13]. It may not be ‘legal representation’ per se that is associated with RTW but these other factors. In addition, there is a lack of granularity when classifying exposure to legal representation. For example, the ‘no win, no fee’ legal services in NSW CTP and WC schemes provide a financial incentive for plaintiff lawyers to take viable cases where extracting a reasonable fee is more likely (e.g. people with more serious injuries, pre-existing and/or crash related factors that could allow access to greater financial entitlements) [45].

### Characteristics of return to work

Measuring RTW is challenging – definitions and durations are diffuse [7]. In our study RTW was measured at three time periods inclusive of time (days) to RTW, full/part-time hours and full/modified duties. At each period the majority were working full-time on full duties but below baseline figures. In similar studies RTW varied from 28-68 % at 6 months [3, 4]; 42 % at 12 months [4]; and 51 % at 24 months [4]. It is difficult to compare RTW rates due to heterogeneity between populations and the multi-dimensional nature of facilitators and barriers for RTW [9].

Taking into account the unemployment rate in Australia over the follow up period (4.2 % in 2008 – 5.4 % in 2013) [46, 47] and the socio-demographic profile of the study population, the limited RTW rate is of concern. Accepting that work is good for health and well-being, the converse is also true and poor health contributes to lost productivity and lower socio-economic status [1].

### Strengths and limitations

This prospective study was a representative cohort of moderate-severe injuries following motor vehicle related orthopaedic trauma. Standardised and validated measures were used with repeated follow up.

Additional measures at baseline would have been beneficial including initial pain intensity, baseline mental health, and other psychological measures. These have been associated with poorer outcomes following trauma [3, 4, 16, 17]. Further, there appears to be a relationship between these factors and having a compensation claim [42, 44]. The inclusion of medical and/or vocational interventions, individual job characteristics/tasks, and workplace/organisational factors would have been useful. These determinants of RTW are often population specific and amenable to intervention in a compensation setting [6, 9].

Another limitation was moderate loss to follow up. The study population characteristics were a plausible reason for loss to follow up. Participants were predominantly younger males of lower socioeconomic status who were in semi-unskilled occupations. They were often contactable (see Fig. 1) but would not return the questionnaires. Those lost to follow up were younger, less likely to be married, and less likely to be currently taking medication. If they had remained in the study, these differences could have influenced time to RTW. Lastly, these findings require validation in future research with larger cohorts and different study populations.

### Future research and policy implications

Predictors of RTW are multidimensional and cover numerous individual, work, organisational and societal domains, which makes high quality research challenging. Despite the abundant research to date, much remains inconclusive [7]. It is important to focus on factors amenable to intervention. Injury severity, pre-injury work hours and health status are relatively static outside the bounds of injury prevention programs.

However, expectations for return to usual activities, illness perception and self-efficacy are more dynamic. Validated measures are now available to gauge this risk factor of poor RTW and/or recovery [37, 39, 48]. Investing in interventions such as education, coaching or multidisciplinary programs could improve RTW rates by adjusting expectations; thereby reducing the associated costs of lost productivity [49–51].

There is a need to understand the paradigm of legal representation, and whether it is a valid measure. Measurement error can occur when the timing of exposure to a factor does not occur at baseline and/or there is questionable quality of the measure [52]. Since legal representation was measured at six months, not baseline, these results need to be interpreted cautiously. Further scheme specific, qualitative and quantitative research – principally of populations at risk for poor RTW – may assist to tease apart these complexities and provide researchers with ideas for RTW initiatives and scheme policy makers with opportunities for legislative or policy change if appropriate.

Lastly, taking into account the significance of lower occupational skill levels, it is crucial to improve RTW rates, and this is feasible, considering the strong evidence base for vocational rehabilitation. The coordination of early work-focused health interventions and accommodating workplaces with modified duties and hours is essential [53]. In WC jurisdictions this is not unforeseen, but in the CTP arena it remains arduous. There is often no legal impetus on the employer to re-employ an injured worker. In this instance, it may be necessary to advocate for legislative change or other policy initiatives like early identification and referral to vocational rehabilitation, or proactive claims management involving the employer to provide appropriate duties in the early post-injury period [53].

## Conclusions

A longer time to RTW was associated with greater injury severity and lower occupational skill levels. A shorter time to RTW was associated with recovery expectations for usual activities of ≤90 days, full-time pre-injury work hours, and very good self-assessed pre-injury health status following motor vehicle related orthopaedic trauma. Our findings reinforce existing research. There is an opportunity to trial interventions that address potentially modifiable factors such as poor recovery expectations. The issues surrounding legal representation are complex and require further research.

## Ethics approval and consent to participate

The study was approved by the governing human research ethics committees (South Western Sydney Local Health District, South Eastern Sydney Local Health District, and The University of Sydney).

## Consent for publication

Not applicable.

## Availability of data and materials

Results from the dataset are presented in the paper. The full dataset is available from the first author upon request.

## Abbreviations

ABS: Australian Bureau of Statistics
AIS: abbreviated injury scale
ASCED: australian standard classification of education
ASCO: Australian standard classification of occupations
AU: Australian dollar
AUDIT-C: alcohol use disorders identification test: self-report version
BEACH: bettering the evaluation of care and health
BMI: body mass index
CALD: culturally and linguistically diverse
CI: confidence interval
CTP: compulsory third party
HILDA: household, income and labour dynamics in australia
HRR: hazards rate ratio
IRSD: index of relative socioeconomic disadvantage
ISS: injury severity score
NHMRC: National Health and Medical Research Council
NISS: new injury severity score
NSW: New South Wales
PCS: physical component score
RTW: return to work
SD: standard deviation
SEIFA: socio-economic indexes for areas
SF36v2: short form-36 version 2.0
WC: workers compensation
