# Erratum to: Predictors of return to work following motor vehicle related orthopaedic trauma

**DOI:** 10.1186/s12891-016-1065-0

**Published:** 2016-05-25

**Authors:** Darnel F. Murgatroyd, Ian A. Harris, Yvonne Tran, Ian D. Cameron

**Affiliations:** John Walsh Centre for Rehabilitation Research, The University of Sydney, Kolling Institute, Sydney, NSW Australia; South Western Sydney Clinical School, UNSW Australia, Liverpool, Sydney Australia; Ingham Institute for Applied Medical Research, Sydney, Australia; South Western Sydney Local Health District, Liverpool, Sydney Australia

## Erratum

After publication of the original article [1], the corresponding author (DF Murgatroyd) noticed that her name was inadvertently duplicated at the beginning and end of the author list. Similarly, the affiliation of this author (John Walsh Centre for Rehabilitation Research, The University of Sydney) had been duplicated as affiliations 1 and 5. The original article has been updated to correct these errors.
